# Temporal Bone Fractures and Related Complications in Pediatric and Adult Cranio-Facial Trauma: A Comparison of MDCT Findings in the Acute Emergency Setting

**DOI:** 10.3390/tomography10050056

**Published:** 2024-05-10

**Authors:** Romain Kohler, Marcella Pucci, Basile Landis, Pascal Senn, Pierre-Alexandre Poletti, Paolo Scolozzi, Seema Toso, Minerva Becker, Alexandra Platon

**Affiliations:** 1 Division of Radiology, Diagnostic Department, Geneva University Hospitals, University of Geneva, 1205 Geneva, Switzerland; romain.kohler@groupe3r.ch (R.K.); marcella.pucci@hug.ch (M.P.); pierre-alexandre.poletti@hug.ch (P.-A.P.); seema.toso@hug.ch (S.T.); minerva.becker@hug.ch (M.B.); 2 Division of Otorhinolaryngology, Head and Neck Surgery, Department of Clinical Neurosciences, Geneva University Hospitals, 1205 Geneva, Switzerland; basile.landis@hug.ch (B.L.); pascal.senn@hug.ch (P.S.); 3 Division of Oral and Maxillofacial Surgery, Department of Surgery, Geneva University Hospitals, University of Geneva, 1205 Geneva, Switzerland; paolo.scolozzi@hug.ch

**Keywords:** cranio-facial trauma, temporal bone trauma, emergency radiology, CT

## Abstract

Purpose: The purpose of this study was to analyze the prevalence of and complications resulting from temporal bone fractures in adult and pediatric patients evaluated for cranio-facial trauma in an emergency setting. Methods: A retrospective blinded analysis of CT scans of a series of 294 consecutive adult and pediatric patients with cranio-facial trauma investigated in the emergency setting was conducted. Findings were compared between the two populations. Preliminary reports made by on-call residents were compared with the retrospective analysis, which was performed in consensus by two experienced readers and served as reference standard. Results: CT revealed 126 fractures in 116/294 (39.5%) patients, although fractures were clinically suspected only in 70/294 (23.8%); *p* < 0.05. Fractures were longitudinal, transverse and mixed in 69.5%, 10.3% and 19.8% of cases, respectively. Most fractures were otic-sparing fractures (95.2%). Involvement of the external auditory canal, ossicular chain and the osseous structures surrounding the facial nerve was present in 72.2%, 8.7% and 6.3% of cases, respectively. Temporal bone fractures extended into the venous sinuses/jugular foramen and carotid canal in 18.3% and 17.5% of cases, respectively. Vascular injuries (carotid dissection and venous thrombosis) were more common in children than in adults (13.6% versus 5.3%); however, the observed difference did not reach statistical significance. 79.5% of patients with temporal bone fractures had both brain injuries and fractures of the facial bones and cranial vault. Brain injuries were more common in adults (90.4%) than in children (63.6%), *p* = 0.001. Although on-call residents reliably detected temporal bone fractures (sensitivity = 92.8%), they often missed trauma-associated ossicular dislocation (sensitivity = 27.3%). Conclusions: Temporal bone fractures and related complications are common in patients with cranio-facial trauma and need to be thoroughly looked for; the pattern of associated injuries is slightly different in children and in adults.

## 1. Introduction

Craniofacial trauma of varying degrees of severity is common. It is typically caused by motor vehicle accidents, sports-related accidents, and interpersonal violence. Concomitant injuries occur in about 16–35% of cases, and they include skull fractures, intracranial hemorrhage and blunt cerebrovascular injury, cervical spine fractures and airway and cutaneous injuries [1,2,3]. Assessments of airways and bleeding, the patient’s neurological status and clearance of the cervical spine are the main priorities in an acute emergency setting, followed by surgical fracture reduction and fixation whenever necessary [2,3]. In addition, patients with cranio-facial trauma can also have concomitant temporal bone fractures, which can lead to major clinical complications and long-term impairment if undetected [4]. The temporal bone houses vital structures related to hearing, balance and maintaining the position of the body, and several other important structures are located within or in close vicinity to the temporal bone (the internal carotid artery, sigmoid sinus and jugular bulb, facial nerve and dura mater).

CT is the imaging modality of choice in both cranio-facial and temporal bone trauma. However, the dedicated CT protocol for temporal bone imaging differs from the protocol used to assess cranio-facial trauma as it requires even higher-resolution thin-slice reconstructions with additional dedicated planes, e.g., the plane of the lateral semicircular canal (axial), Stenvers’ plane (coronal oblique) and the plane of Pöschl (sagittal oblique), and several authors recommend the use of such a dedicated protocol whenever temporal bone fractures are suspected. Other tailored planes obtained along certain structures, e.g., along the stapes or parallel to the facial nerve canal, also play an important role in diagnosis as they facilitate the detection of lesions specifically involving these structures. Since the introduction of MDCT scanners (64-slice and above), high-resolution thin-slice volume acquisitions have enabled multi-planar reconstructions (MPRs) in any plane and the 3-D visualization of structures, thus leading to a dramatic improvement in the detection of temporal bone injuries and complications.

Temporal bone trauma presents unique diagnostic challenges in both adults and children due to anatomical differences, the size of middle ear structures and possibly different trauma mechanisms. Pediatric temporal bones are more pliable and cartilaginous compared to adult bones, which are denser and more ossified. This can theoretically influence fracture patterns, clinical presentation and the extent of injury. According to many authors, in adults, temporal bone fractures often result from high-velocity trauma such as motor vehicle accidents. However, in children, temporal bone fractures are commonly associated with falls or sports-related injuries. Likewise, clinical presentation may differ in adults and children: children may have more subtle symptoms or nonspecific signs of temporal bone trauma compared to adults, potentially leading to a delay in diagnosis, whereas adults may present with classic signs such as Battle’s sign (bruising over the mastoid process) or hemotympanum. Some authors, however, reported that children do not differ from adults in terms of trauma mechanism, fracture type or associated injuries [5,6,7]. In addition, to the best of our knowledge, only one study so far has compared temporal bone trauma patterns and associated injuries in adults versus children [5]. 

The objectives of this study were first to evaluate the incidence and types of temporal bone fractures and associated injuries in patients with craniofacial trauma seen in an emergency setting. Second, we aimed to compare temporal bone injuries in adults versus children and third, we compared the diagnostic performance of initial CT evaluations performed by on-call radiology residents with evaluations performed by a specialist head and neck radiologist. To best of our knowledge, no other study has addressed this topic thus far.

## 2. Materials and Methods

This retrospective study was approved by the Institutional Ethics Committee and performed in accordance with the guidelines of the Helsinki II Declaration. Informed consent was waved.

Our institution is a university trauma center with a 24 h on-call unit covering a referral area of approximately 500,000 and with 50,000 emergency visits per year. Inclusion criteria were adult and pediatric patients with blunt or penetrating craniofacial trauma having undergone a CT examination in an emergency setting; a further analysis identified patients with a radiological diagnosis of temporal bone fracture. We performed a computerized search of the PACS archives and medical records of our institution for a period of 5 consecutive years and identified 294 consecutive head and facial trauma patients who underwent MDCT of the head and face with a maximum reconstructed slice thickness of 1 mm. Among the 294 patients, 168 patients did not have temporal bone fractures, whereas 116 patients did (prevalence of temporal bone fractures = 116/294 (39.5%)). The gold standard for the diagnosis of temporal bone fractures consisted of a CT assessment by two senior head and neck radiologists and, in addition, clinical, audiometric and otoscopic assessments by senior otorhinolaryngologists. The 116 patients with temporal bone fractures form the basis of the current study. There were 40 females (mean age = 45.0 years) and 76 males (mean age = 36.4 years) and 94 adults (mean age = 39.4 years) and 22 children (mean age = 7.7 years). 

Falls comprised the most frequent mechanism of trauma (n = 61, 52.6%). The other mechanisms included motor vehicle accidents (n = 34, 29.3%), direct blows (n = 19, 16.4%) and gunshot injuries (n = 2, 1.7%). Regarding trauma mechanisms in children and adults, in both groups, falls were the most common causes of temporal bone fracture (49/94, 52%, in adults versus 12/22, 54.5%, in children), followed by motor vehicle accidents (28/94, 29.8%, in adults versus 6/22, 27.3%, in children); *p* > 0.05. Thirty-seven patients (37/116, 31.9%) had tracheal intubation at the time of CT, thus impairing symptom evaluation. Otorrhagia/hemotympanum was the most frequent clinical finding (n = 57, 49%) in intubated and non-intubated patients, followed by hearing loss (n = 8, 6.8%), facial nerve palsy (n = 4, 3.4%) and otorrhea (n = 1, 0.8%). 

### 2.1. CT Protocols

All 116 patients underwent an MDCT examination (Philips MX 8000, Philips Medical Systems, Best, The Netherlands, 16 detectors and 64 detectors) with a reconstructed slice thickness of 0.4–1 mm on the temporal bone and with or without an intravenous injection of iodinated contrast material. Twenty-one patients underwent an additional total body scan, and the remaining 95 patients underwent an additional head CT scan. 

### 2.2. Two-Dimensional MPR

All examinations were reviewed retrospectively using our routine PACS workstations, using OsiriX imaging software (OsiriX v4.0 64 bits; Pixmeo, Geneva, Switzerland). With the aim of having a standardized analysis protocol, each temporal bone was reconstructed in real time in a 2D MPR. The slice thickness for the real-time 2D MPR reconstructions was 0.6 mm, with a reconstruction interval of 0.5 mm using a bone algorithm. The axial plane was defined as the plane of the lateral semi-circular canal. The coronal plane was perpendicular to the plane of the lateral semi-circular canal. When needed, other oblique planes were reconstructed (for example, the axis of the incudo-malleolar joint or perpendicular to the petrous apex).

### 2.3. CT Interpretation and Data Collection

Both temporal bones of each patient (n = 232 temporal bones) were interpreted in consensus by two radiologists with 8 years and 15 years of experience in head and neck radiology and without knowledge of the clinical data or follow-up results. All findings were recorded. The main type of fracture was determined first. If the main fracture line coursed along the long axis of the pyramid of the temporal bone, it was defined as a longitudinal fracture. If the main fracture line was located along the short axis (perpendicular to the long axis), it was a transverse fracture. Complex fractures were considered a combination of longitudinal and transverse fractures. The analysis also included the radiological pattern of complications of the temporal bone fractures: otic or extra-otic extension and the involvement of the tympanic cavity, ossicular chain, facial canal, cochlea, vestibule, semicircular canals, carotid canal, the region of the sigmoid sinus and jugular foramen, the external auditory canal and temporomandibular joint and the petrous apex. The patency of arterial and venous structures was noted (dissection or thrombosis, respectively) in patients using an intravenous iodine injection of contrast medium. Associated injuries, defined as face fractures, cranial vault fractures and concomitant intracranial injuries were also recorded.

### 2.4. Analysis from Radiologic Reports

To determine the quality of the initial CT evaluation, we checked the medical records to assess the preliminary evaluations of the on-call residents (3rd–5th year residents) and recorded which fractures were seen, suspected or overlooked. We then compared these prospective findings to results of our retrospective analysis. 

### 2.5. Statistical Analysis

The data were analyzed using a dedicated statistical software (QuickCalcs©2024, GraphPad Software, Boston, MA, USA). Descriptive statistics and proportions were calculated. A χ^2^ test was performed to assess the differences between categorical variables. The level of statistical significance for all tests was defined as *p* < 0.05.

## 3. Results

### 3.1. Temporal Bone Fractures

Of the 116 patients with temporal bone fractures, 10 patients (8.6%) had bilateral fractures, thus resulting in a total of 126 temporal bone fractures. Among the 126 fractures, the left (68/126) and right (58/126) sides were almost similarly distributed, with a slight predominance of the left (54%).

**Figure 1:**
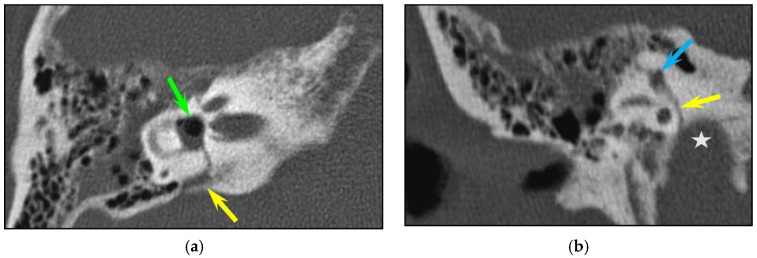
Inner ear injuries. A transverse fracture in an 84-year-old woman with otorrhagia after a fall. CT of the right temporal bone; (**a**) axial plane; (**b**) coronal plane. (**a**) Axial plane: The fracture line extends inferiorly from the aqueduct of the vestibule (yellow arrow) to the vestibule, with associated pneumolabyrinth (green arrow). (**b**) The fracture line (yellow arrow) also involves the jugular foramen (asterisk) and the superior semicircular canal (blue arrow).

Longitudinal fractures were the most frequent pattern (88/126, 69.4%), followed by transverse (13/126, 10.3%) and complex (25/126, 19.8%) types. Only six fractures (6/126, 4.8%) showed involvement of the otic capsule. In one case, there was no visible fracture line but only pneumolabyrinth (in the vestibule and in the superior semi-circular canal). Three patients had a combination of fracture line and pneumolabyrinth (pneumovestibule: n = 3; air in semi-circular canals (n = 2) (Figure 1)).

**Figure 2:**
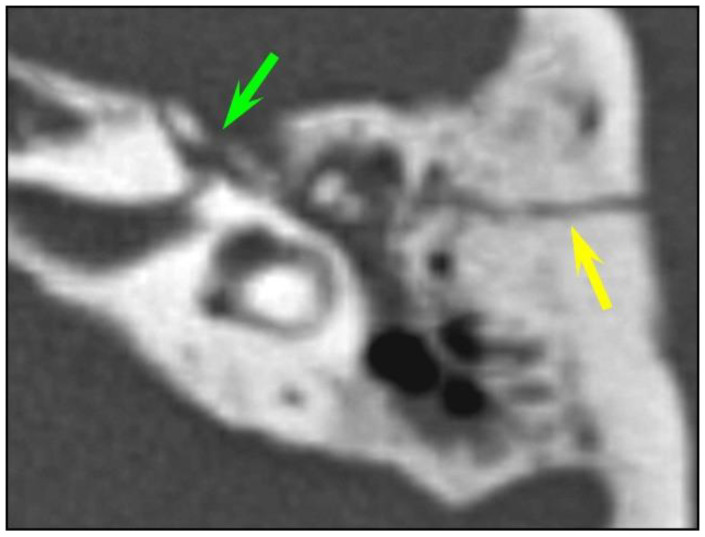
Facial canal injuries. Fracture of the left temporal bone in a 25-year-old man with peripheral facial palsy after a fall. CT of the left temporal bone: longitudinal fracture (yellow arrow) with associated bony fragments in the geniculate ganglion fossa (green arrow) consistent with a geniculate facial canal injury.

In total, fractures involved the bony labyrinth in 11 cases and extended into the following structures: semi-circular canals (posterior semi-circular canal, n = 3; superior semi-circular canal, n = 2), vestibule (n = 3), cochlea (n = 1) and the vestibular aqueduct (n = 2). Involvement of the internal auditory canal was seen in two cases. Fracture lines extended into the petrous apex in 10/126 (7.9%) of all temporal bone fractures, and fractures along the osseous structures surrounding the facial nerve were seen in 8/126 (6.3%) cases. The fractures lines involved the geniculate fossa in four cases (Figure 2), the facial nerve canal in the tympanic cavity in two cases and the bony structure surrounding the second genu of the facial nerve in two cases, respectively. Air in the tympanic facial canal without a visible fracture line was present in one case.

**Figure 3:**
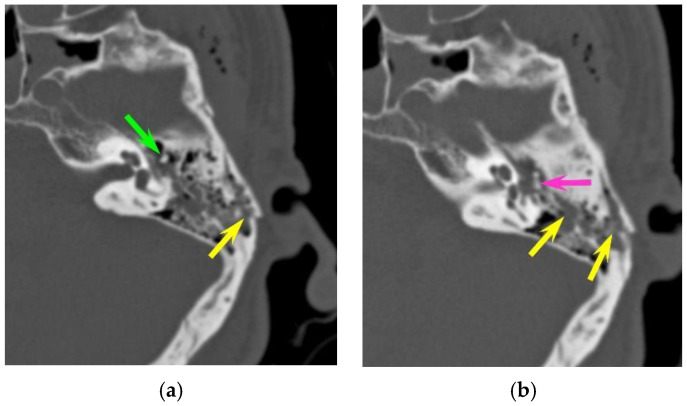
Ossicular chain dislocation. A polytrauma patient with otorrhagia. CT of the left temporal bone (axial plane). (**a**,**b**) Longitudinal fracture (yellow arrow); the malleus head (green arrow) is displaced and isolated from the incus body (pink arrow), corresponding to an incudo-malleolar luxation.

Eleven temporal bone fractures (11/126, 8.7%) showed involvement of the ossicular chain. The incudo-mallear joint was the most frequently injured joint (n = 6), displaying luxation or subluxation (Figure 3). Other injuries were complete incus dislocation (n = 3) and incudo-stapedial luxation (n = 2). Fractures of the tympanic cavity walls were much more common (67/126, 53.2%) than ossicular dislocations. Likewise, fractures of the external auditory canal–temporomandibular joint complex were more common than ossicular chain injuries (91/126, 72.2%).

**Figure 4:**
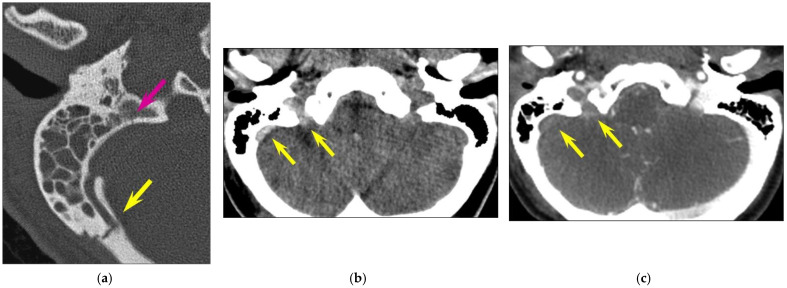
Vascular injuries. Longitudinal fracture of the right temporal bone and venous thrombosis in a 30-year-old woman involved in a motorway traffic accident. CT of the posterior fossa (**a**,**b**) unenhanced CT; (**c**): intravenous contrast-enhanced CT. (**a**) A detached fragment of the posterior part of the temporal bone, projecting into the sigmoid sinus (yellow arrow); the fracture line is also seen more medially (pink arrow). (**b**): spontaneous hyperdensity of the sigmoid sinus and of the jugular foramen (yellow arrows). (**c**) The absence of the opacification of these vascular structures, consistent with venous thrombosis (yellow arrows).

In 22 fractures (22/126, 17.5%), the fracture line involved the carotid canal, but only one case (1/126, 0.8% of all temporal bone fractures; 1/22, 4.5% of fractures involving the carotid canal) showed associated focal internal carotid artery dissection. In 23 cases (23/126, 18.3%), the fracture line extended to the sigmoid groove of the petrous temporal bone; thrombosis of the sigmoid sinus (Figure 4) was present in 4 cases (4/126, 3.2% of all temporal bone fractures; 4/23, 17.4% of fractures involving the sigmoid groove).

### 3.2. Associated Injuries

As many as 92/116 (79.3%) of the patients with temporal bone fractures also had associated fractures of the face or cranial vault. Temporal bone fractures were most often associated with fractures of the parietal bones (27/116, 23.2%), occipital bones (28/116, 24.1%) and sphenoid (31/116, 26.7%). Less often, they were associated with midfacial fractures, i.e., naso-orbitoethmoid or orbital fractures (22/116, 18.9%) and zygomatic arch fractures (6/116, 5%), while associations with isolated maxillary bone or mandibular condylar fractures were seen only in 1/116 (<1%) and 1/116 (<1%) of patients, respectively. In temporal bone fractures, the squamous portion of the temporal bone was most frequently affected (60/92, 65.2%). Most patients with temporal bone fractures showed intracranial associated injuries (99/116, 85.3%) which included the following: epidural hematoma (76/116, 65.5%), subdural hematoma (55/116, 47.4%), subarachnoid hemorrhage (64/116, 55.2%), brain hematoma including diffuse axonal injury (70/116, 60.3%), intraventricular hemorrhage (12/116, 10.3%), brain oedema (20/116, 17.2%), vascular complications (8/116, 6.9%: 7 sigmoid sinus thrombosis and 1 internal carotid artery dissection), brain herniation (18/116, 15.5%), pneumocranium and hematoma (61/116, 52.6%). Only 5/116 patients had isolated pneumocranium (4.3%).

### 3.3. Pediatric Patients

Our patient series comprised 22 (19%) children (defined as age < 16 years). Even if the sizes of the adult and pediatric populations were different (94 and 22, respectively), the incidences of ossicular chain dislocation (3/22, 13.7%, in children versus 8/104, 7.7%, in adults) and of vascular injuries (13.6% in children versus 5.3% in adults) appeared to be higher in children, though without reaching statistical significance, *p* > 0.05. The incidence of associated fractures of the face and cranial vault (15/22, 68.2%, in children versus 77/94, 81.9%, in adults) was similar in both groups, *p* > 0.05. However, children showed associations with brain injuries less often than adults (14/22, 63.6%, in children versus 85/94, 90.4%, in adults), *p* = 0.001 (Table 1). In children with temporal bone fractures, additional fractures involved the parietal bones (2/22, 9.1%), the frontal bones (1/22, 4.5%), the occipital bones (5/22, 23%) and the sphenoid bone (2/22, 9.1%). 

### 3.4. Analysis of Radiologic Reports

An electronic preliminary report written by the on-call resident was available for 111 fractures. In five cases, only a definitive report by the attending on call staff member was available. Among the preliminary reports written by on-call residents, temporal bone fractures were missed in 3 cases (2.7%), suspected in 5 cases (4.5%) and correctly diagnosed in the remaining 103 cases (92.8%). Ossicular dislocation was missed in 8/11 (72.7%) dislocations and the extension of the fracture line into the facial canal was missed in 4/8 (50%). 

## 4. Discussion

The advent of MDCT led to a paradigm shift in the imaging of skull base trauma and, since then, several classifications of temporal bone fractures have been described with variable clinical correlations [8,9,10]. In this study, we used two classifications as references: (a) fractures classified as longitudinal, transverse or mixed, which corresponds to the classification most commonly used in clinical practice (b) fractures classified as involving the otic capsule and sparing the otic capsule, a classification which—according to some authors—predicts sensorineural loss, vestibular dysfunction, facial nerve injury, internal carotid artery injury and CSF leaks [11,12,13].

Only a few studies have examined temporal bone fractures in children [14,15,16] so far and, to our knowledge, only one study has compared adult and pediatric populations [5]. As in the study of Kang et al., we found no difference regarding trauma mechanisms in children versus adults, with falls comprising the most common mechanism, followed by motor vehicle accidents [5]. 

In our study, ossicular chain injuries were slightly more common in children (13.7%) than in adults (7.7%), and the incudo-mallear joint was the most frequently injured joint in both groups, followed by incus dislocation and incudo-stapedial luxation. Similar results were seen in a case series by Meriot et al. which showed that incudo-mallear joint dislocation was the most common ossicular abnormality (59%), followed by incudo-stapedial joint dislocation (53%) and incus dislocation [17]. As pointed out by different authors, ossicular dislocation can be very subtle. Subluxation of the incus without obvious displacement and with only a minor increase in the space between the articulation surfaces of the incus and malleus can be easily missed on an initial CT scan obtained in an emergency situation. Likewise, the diagnosis of incudo-stapedial luxation can be easily missed unless dedicated 2D oblique reconstructions are obtained. The presence of hemotympanum further hampers ossicular evaluation, especially of the stapes superstructure due to the very small size of the two crura. In our study, most ossicular chain injuries (72.7%) were missed by the resident on call; however, they were detected at a retrospective CT evaluation by specialized head and neck radiologists. Patients with ossicular dislocation typically present with conductive hearing loss persisting several months or years after temporal bone trauma, and the reported delay between the initial injury and diagnosis followed by treatment is within a range of 5–6 years [18]. As post traumatic ossicular dislocation also occurs with head trauma without temporal bone fracture, ossicular disruption should be suspected in all patients with conductive hearing loss persisting after 2-months of the healing process [18]. In these patients, a high-resolution CT is recommended to facilitate diagnosis as the hemotympanum usually resolves 2 months after trauma. Fractures of the malleus, incus or stapes are exceedingly rare and tend to occur after digital manipulation of the external auditory canal or from torsional injuries. As expected, we did not detect any ossicular fractures in this series.

Otic capsule fractures were rare in our series (4.8%). These data are confirmed by the literature, with a prevalence of around 5% in most series (2.5% [11], 5.6% [13], 5.8% [10], 7% [9], and 18.6% [8]. Pneumolabyrinth is the second important sign and can sometimes present isolated, as in one of our cases. Pneumolabyrinth typically presents as air bubbles within the vestibule, semi-circular canals or cochlea. It is considered a sign of a traumatic perilymphatic (labyrinthine) fistula which corresponds to an abnormal communication between the fluid-filled inner ear and the air-filled middle ear. Although patients usually present with sensorineural hearing loss, vertigo or tinnitus, perilymphatic fistula is often overlooked upon initial clinical examination due to the predominance of neurological symptoms in the emergency situation [19]. As perilymphatic fistulae can decompensate by efforts with increased pressure, early detection, patient counseling and/or surgical intervention are essential to avoiding irreversible hearing loss. Data regarding the true prevalence of pneumolabyrinth in temporal bone trauma are scarce and contradictory; some authors have suggested that it may occur in 8% of all temporal bone fractures and in 50% of otic capsule fractures [20]. However, a review of the international literature from 2014 found only 14 reported cases of pneumolabyrinth, most of which were associated with transverse temporal bone fractures (10/14 cases), and the authors concluded that pneumolabyrinth in the setting of temporal bone trauma is a rare phenomenon [21]. In our series, all otic capsule fractures had associated pneumolabyrinth and, in one case, it was only the presence of pneumolabyrinth—without a clearly visible fracture line—that suggested otic capsule involvement. 

Several studies also showed the high prevalence of traumatic brain injuries related to temporal bone fractures (30.4% [22], 58% [23] and 91.1% [24]). As fractures violating the otic capsule are probably the consequence of a more energetic trauma, this type of fracture seems to be associated with more injuries elsewhere [13]. In a series of pediatric temporal bone fractures (n = 323), Waissbluth et al. [7] reported intracranial hemorrhage in 62% of patients, which included subarachnoid (21.3%), subdural (21.3%) and epidural hemorrhages (19.6%).

Our results (85.3% with traumatic brain injuries and 79.3% with fractures of the face and cranial vault) match with the results of the aforementioned studies. Epidural (65.5%) and subdural (47.4%) hematomas were the most frequent extra-axial traumatic brain injuries, but intra-axial hematoma was the leading injury (60.3%). With 15.5% of brain herniation and 17.2% of brain edema, our study emphasizes that a significant number of patients with temporal bone fractures may need immediate neurosurgical work-up. It is worthwhile mentioning that—in our series—brain injuries were more common in the adult population compared to the pediatric population despite similar trauma mechanisms.

A study found that 3.4% of head injuries were associated with petrous temporal bone fractures [22]. The reported incidence of dural venous thrombosis in association with temporal bone fracture, more so with those crossing the dural sinus or jugular bulb, is about 40.7% [25]. When the fracture involves the petrous portion of the temporal bone, the reported incidence of dural venous thrombosis is 22.4% [26]. In our study, the incidence of dural venous thrombosis was 17.4% in fractures involving the sigmoid groove of the petrous temporal bone. It is worth mentioning that no venous thrombosis was detected via CT whenever the fracture line did not involve the sigmoid groove. We had also one internal carotid dissection in an adult (0.8% of all temporal bone fractures and 4.5% of fractures involving the carotid canal). Internal carotid artery injury is reported to occur in 1% of all adult patients with severe head trauma [27] and in up to 9.4% of patients with petrous temporal bone fractures [28]. Complications include hemorrhage, pseudoaneurysm formation, carotid cavernous fistula, stroke and death, with a reported in-hospital mortality rate of up to 45.5% [28]. According to some authors, in adult patients with petrous temporal bone fractures, factors clearly associated with internal carotid artery injury include carotid canal fractures and motor vehicle or motorbike crashes as trauma mechanisms [28]. As these patients have an increased risk of internal carotid injury, some authors have recommended routine screening for internal carotid artery injury using CT angiography [28]. Internal carotid artery injury is only rarely seen in the pediatric population, and data are limited to isolated case reports. Nevertheless, for cranial trauma in children, fractures of the petrous temporal bone or through the carotid canal have been shown to be independent factors for blunt cerebrovascular injury [29]. 

Facial nerve palsy is a major clinical concern in the event of temporal bone fracture, and it has been reported to occur in about 7–10% of fractures. In most cases, the onset of facial nerve palsy is immediate; however, in a minority of cases, facial nerve palsy may present with delayed onset (after 5 days) due to inflammation and swelling compressing the facial nerve. In our study, radiologically detected injuries of the facial canal and the osseous boundaries of the other facial nerve segments were present in eight cases, most often in the region of the geniculate ganglion; however, facial palsy was present or developed later in only four cases. The incidence of facial nerve palsy was, therefore, very low (only 3%), which is in contradiction to other data from the literature, in which the reported incidence of complete facial palsy is quite high: 25% in longitudinal fractures and 44% in transverse fractures, respectively [11]. In some cases, patients develop facial palsy without a visible fracture line, as in one of our cases, in which only a tiny air bubble was present in the facial canal. It is therefore possible that some fractures are not detectable with a standard CT protocol, as conducted in an emergency setting. In these cases, additional evaluation with a dedicated high-resolution CT scan of the temporal bone is indicated if decompression of the facial canal is planned in case of post-traumatic facial nerve palsy [30,31].

In the temporal bone, many intrinsic fissures, extrinsic fissures and intrinsic channels exist and may lead to some diagnostic difficulty [32]. However, with a thorough examination of images and good knowledge of anatomy, all these potential pitfalls may be easily avoided.

The residents on call were able to reliably detect temporal bone fractures in most instances (92.8%). However, the identification of a “temporal bone fracture” alone is not sufficient for further clinical management, which also requires describing the type of fracture (longitudinal, transverse or mixed) and assessing the integrity of the otic capsule and ossicular chain via CT. As shown in the results section, more subtle findings, such as ossicular dislocation or facial canal involvement, were often overlooked in the initial report. To increase the detection rate of ossicular dislocation or facial canal involvement, dedicated 2D oblique reconstructions or even 3D reconstructions are very helpful [33]. Orman showed that 3D CT reconstructions significantly improved the skull fracture detection rate in pediatric patients. 

Our study has several drawbacks. First, it was a retrospective, single-center study, and we enrolled patients based on a computerized search of the PACS archives and medical records of our institution using keywords. It is therefore possible that the real prevalence of temporal bone fractures may be underestimated as some overlooked fractures would not have been mentioned in the records. Similarly, as we excluded all CT examinations with slices exceeding 1 mm of thickness, there is a possibility that some fractures were not taken in account.

Second, the aim of the present study was to depict the various extensions of temporal bone fractures, including the involvement of adjacent structures. Therefore, initial and long-term clinical assessments were not examined here. 

The mechanisms of injury in our study may be different from other study groups, which may have impacted our results.

## 5. Conclusions

Temporal bone fractures and related complications are common in patients with cranio-facial trauma and need to be thoroughly looked for. The pattern of associated injuries is slightly different in children and adults despite similar trauma mechanisms, which mainly include falls and motor vehicle accidents. While brain injuries are more common in adults, ossicular dislocation and vascular injuries tend to be more common in children, though without reaching statistical significance. On-call residents achieve a high level of sensitivity for the detection of temporal bone fractures in an emergency setting; however, more subtle lesions, such as ossicular dislocations and involvement of the facial canal, are often missed. Evaluations by senior radiologists and with multiplanar oblique 2D reconstructions are necessary to improve the detection of these associated lesions.

## Figures and Tables

**Table 1:** Temporal bone fractures and associated injuries in adult and pediatric patients.

	Adult Patients n = 94 (%)	Pediatric Patients n = 22 (%)	*p*-Value
Vascular injuries	5 /94 (5.3%)	3/22 (13.7%)	0.16
Face and cranial vault fractures	77/94 (81.9%)	15/22 (68.2%)	0.15
Brain injuries	85/94 (90.4%)	14/22 (63.6%)	0.001

## Data Availability

The data are not publicly available due to the institutional ethics committee’s privacy restrictions.
